# A Novel UHPLC-MS/MS Method for the Quantification of Seven Opioids in Different Human Tissues

**DOI:** 10.3390/ph16060903

**Published:** 2023-06-19

**Authors:** Alessandra Manca, Amedeo De Nicolò, Elisa Delia De Vivo, Micol Ferrara, Sharon Oh, Sahar Khalili, Niamh Higgins, Robert G. Deiss, Stefano Bonora, Jessica Cusato, Alice Palermiti, Jacopo Mula, Sara Gianella, Antonio D’Avolio

**Affiliations:** 1 Laboratory of Clinical Pharmacology and Pharmacogenetics, Department of Medical Sciences, University of Turin, Amedeo di Savoia Hospital, Corso Svizzera, 164, 10149 Turin, Italy; alessandra.manca@unito.it (A.M.);; 2 Unit of Infectious Diseases, Department of Medical Sciences, University of Turin, Amedeo di Savoia Hospital, 10149 Turin, Italy; 3 San Diego Division of Infectious Diseases and Global Public Health, University of California San Diego, La Jolla, CA 92037, USA; 4 CoQua Lab s.r.l., 10149 Turin, Italy

**Keywords:** LC-MS, tissue, morphine, fentanyl, opioids

## Abstract

Background: Opioids are considered the cornerstone of pain management: they show good efficacy as a first-line therapy for moderate to severe cancer pain. Since pharmacokinetic/pharmacodynamic information about the tissue-specific effect and toxicity of opioids is still scarce, their quantification in post-mortem autoptic specimens could give interesting insights. Methods: We describe an ultra-high-performance liquid chromatography coupled with tandem mass spectrometry method for the simultaneous quantification of methadone, morphine, oxycodone, hydrocodone, oxymorphone, hydromorphone and fentanyl in several tissues: liver, brain, kidney, abdominal adipose tissue, lung and blood plasma. The presented method has been applied on 28 autoptic samples from different organs obtained from four deceased PLWH who used opioids for palliative care during terminal disease. Results: Sample preparation was based on tissue weighing, disruption, sonication with drug extraction medium and a protein precipitation protocol. The extracts were then dried, reconstituted and injected onto the LX50 QSight 220 (Perkin Elmer, Milan, Italy) system. Separation was obtained by a 7 min gradient run at 40 °C with a Kinetex Biphenyl 2.6 µm, 2.1 × 100 mm. Concerning the analyzed samples, higher opioids concentrations were observed in tissues than in plasma. Particularly, O-MOR and O-COD showed higher concentrations in kidney and liver than other tissues (>15–20 times greater) and blood plasma (>100 times greater). Conclusions: Results in terms of linearity, accuracy, precision, recovery and matrix effect fitted the recommendations of FDA and EMA guidelines, and the sensitivity was high enough to allow successful application on human autoptic specimens from an ethically approved clinical study, confirming its eligibility for post-mortem pharmacological/toxicological studies.

## 1. Introduction

Opioids have been the most useful drugs for the management of severe pain for more than 200 years [1]. They show good efficacy as a first-line therapy for moderate to severe cancer pain with greater analgesic efficacy than non-steroidal anti-inflammatory drugs.

These drugs act by binding opioid receptors located along the nociceptive pathway [2] name µ (in turn including µ-1 and µ-2 subtypes), κ and δ [3,4]. Several opioids are available for clinical use in the management of chronic pain, with the most common including morphine (MOR), methadone (MTD), hydromorphone (H-MOR), hydrocodone (H-COD), oxycodone (O-COD), oxymorphone (O-MOR), and fentanyl (FENT) [5,6]. MOR is a phenanthrene derivative and the prototypical µ-receptor opiate [3]; it is the first-line treatment of severe pain and cancer [7]. After oral administration, about 40 to 50% of the administered dose reaches the central nervous system. In small amounts, MOR is also metabolized in H-MOR: this last is present in 66% of MOR consumers without excessive drug response [3,8]. FENT is an opioid agonist that is about 80–100 times more potent than morphine, highly lipophilic and highly bound to plasma proteins [3,9]. H-COD is the most used opioid indicated for moderate to severe pain. O-COD has similarities with H-COD and has activity at multiple opiate receptors including the k-receptor. O-COD has high affinity for the μ receptor and is about 10 times more potent than MOR, and it is not affected by CY2D6 or CY3A4 [3]. H-MOR is more potent than MOR (7–10 times more potent), and it has a good solubility in water which allows for concentrated formulations [10].

MTD is a synthetic μ opioid receptor agonist; in addition to its opioid activity, it is also an antagonist of the N-methyl-D aspartate (NMDA) receptor [3].

The management of these drugs is complex due to their effects on the central nervous system but also on other systems, causing respiratory depression, orthostatic hypotension, constipation, urinary retention, nausea and vomiting [3] and, in most critical cases, coma and death [11].

Most opioids are subjected to wide first-pass metabolism in the liver before reaching systemic circulation. Metabolism allows facilitating renal excretion, improving drug hydrophilicity. CYP450 and UDP-glucuronosyltransferases (UGTs) are the two major enzyme systems involved in opioids metabolism: this process results in the production of both inactive and active products [12].

Some opioids are pro-drugs, and they become active after the metabolism process. Others opioids are transformed in more potent drugs after an initial metabolism [13,14].

As an example, COD is a pro-drug that shows pharmacological activity after metabolism to MOR in the liver. About 80% of COD is eliminated by glucuronization through the UGT enzyme. A minor pathway (6–9% of the dose) is represented by N-demethylation to nor-COD and O-demethylation to MOR by the CYP2D6 enzyme [12].

Indeed, H-COD is metabolized by CYP enzymes. More than 50% of total H-COD clearance is mediated by CYP2D6 and CYP3A4, resulting in the formation of H-MOR and norhydrocodone, respectively. H-COD is metabolized with glucuronization, and it is about 10 times more potent and less polar than its parent drug, codeine. H-COD may be a pro-drug, requiring further metabolism to H-MOR, which is an active opioid agonist [12].

Since pharmacokinetic/pharmacodynamic information about the tissue-specific penetration and toxicity of opioids is still scarce, their quantification in post-mortem autoptic specimens could give interesting insights about the tissue distribution of these drugs.

Some opioids are lipophilic and can be stored in body tissues for a long time: thanks to their good solubility in lipids, some opioids are rapidly distributed in tissues. Consequently, they can be gradually released, causing tissue redistribution [15]. In this scenario, investigation about opioid distribution in tissues could be a useful tool to better understand tissue-specific toxicity: in this scenario, the post-mortem toxicological investigation of different specimens is needed. Consequently, thoroughly validated analytical methods capable of a reliable quantification of these drugs in different tissues are required.

Most tissue distribution studies are performed in animals models [16,17], while poor information is available about distribution in humans [18,19,20]. In addition, the currently available observations in humans are mainly focused on toxicological/forensic qualitative or semi-quantitative applications, serving more than a quantitative purpose in the context of pain management.

Furthermore, a very small variety of matrices are normally evaluated even in the toxicological analysis, such as hair [21], nails [22,23], saliva [24], plasma, blood, urine and, in rare post-mortem analyses, brain or liver [11,25,26,27,28].

The evaluation of these molecules in different biological matrices should be carried out with sensitive and specific methodologies, such as liquid chromatography coupled with mass spectrometry (UHPLC-MS/MS) [29,30]. This technique has been also applied in several fields, for example for the quantification of different phytocostituents and metabolites, as shown in the works of Thakur et al. and Perez De Souza et al. [31,32].

For these reasons, in this work, we reported on the development and validation, following EMA and FDA guidelines [33,34], of an ultra-high performance liquid chromatography coupled with tandem mass spectrometry (UHPLC-MS/MS) method for the simultaneous quantification of MOR, O-COD, H-COD, O-MOR, H-MOR, FENT and MTD in several animal tissues (e.g., liver, brain, kidney, heart, lung) and its application in human autoptic samples. Interestingly, these samples were from a unique cohort of terminally sick participants with concomitant HIV infection who agreed to participate in the study, donating their post-mortem tissues, which were withdrawn and snap frozen within 6 h after death. Furthermore, compared to the literature, the present method is able to quantify a larger number of opioids in different specimen types.

## 2. Results

### 2.1. Calibration Curve and Dilution Integrity

During method validation, all drugs showed linear calibration curves. The coefficient of determination (R^2^) of all calibration curves ranged from 0.996 to 0.999, confirming good fitting to the calibration models. The equations are reported in Table 1.

Samples spiked with concentrations higher than STD 6 were quantified with a mean bias lower than 10% after a 3-fold dilution with extraction solvent, highlighting a good dilution integrity.

### 2.2. Specificity and Selectivity

**Figure 1:**
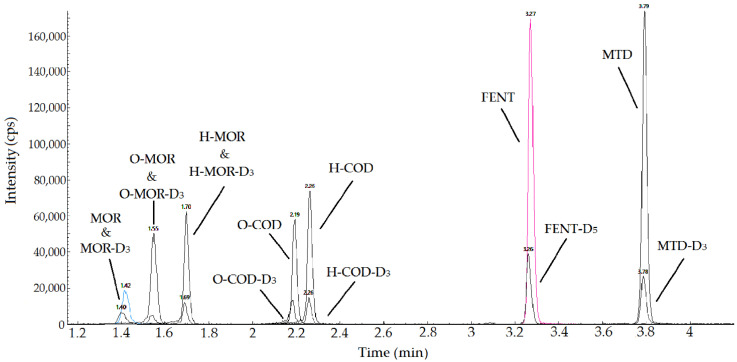
Overlaid chromatograms of the target analytes and their internal standards from the analysis of a medium level of the standard curve (STD 5).

The chromatographic separation of all the analytes and their IS medium standard point (STD 5) have been depicted in Figure 1: a summary of the RT of opioids is provided in Table 2.

**Figure 2:**
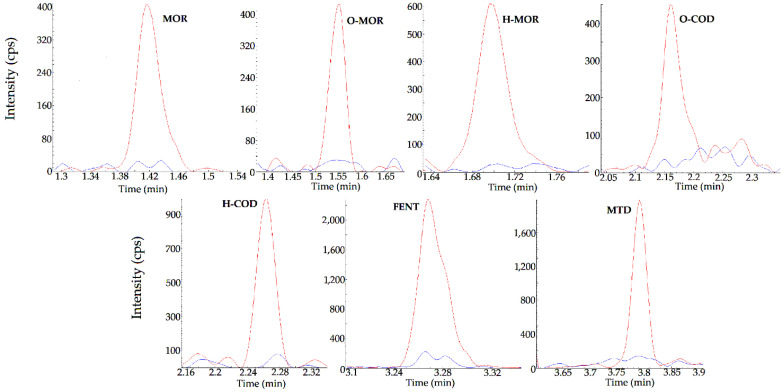
Overlaid chromatograms from the injection of the lowest level of the standard curve (STD 1 = LLOQ) and from blank liver sample (STD 0) for each analyte.

The blank tissues samples did not yield any significant “noise” (20% of the signal of the analytes at the LLOQ or 5% of the IS) due to endogenous components at the analytes’ RT (Figure 2).

Similarly, the addition of antiretroviral drugs (Abacavir, Amprenavir, Atazanavir, Bictegravir, Cabotegravir, Cobicistat, Darunavir, Doravirine, Dolutegravir, Efavirenz, Elvitegravir, Emtricitabine, Etravirine, Lamivudine, Lopinavir, Maraviroc, Nevirapine, Raltegravir, Rilpivirine, Ritonavir, Tenofovir Disoproxil Fumarate and Tenofovir Alafenamide) did not yield significant interference neither in terms of additional signal nor matrix effect (ME).

### 2.3. Lower Limit of Quantification (LLOQ) and Limit of Detection (LOD)

The lower limits of quantification (LOQ) and of detection (LOD) are reported in Table 3.

The LLOQ resulted at least equal to the STD1, as required by FDA guidelines.

The overlaid chromatograms for each analyte at the LLOQ and in blank plasma are reported in Figure 2.

### 2.4. Stability

Long-term stability data showed deviation lower than 15% after 4 months at −80 °C. Similarly, short-term stability bench-top and in an autosampler has resulted in CV% in accordance to reference guidelines for method validation. All drugs were not affected by 2 freeze and thaw cycles, as reported in Supplementary Table S1.

### 2.5. Recovery and Matrix Effect

The recovery (REC) data, both in terms of absolute REC and in terms of IS-normalized REC, were consistent and highly reproducible among different matrices and matrix lots for each analyte. Similarly, the matrix effect (ME) results were quite variable among different tissues: nevertheless, the evaluation of IS-normalized ME (IS-nME) showed the very good performance of the chosen IS compounds to correct the variability accounted by ME, which was in accordance with previous reports and with EMA guidelines [33,37]. Data are summarized in Table 4.

### 2.6. Carry-Over

Carry-over was investigated by injecting blank plasma extracts after the injection of a sample extract prepared at a twice higher concentration than the highest standard sample (STD 6). Resulted data showed the absence of significant carry-over.

### 2.7. Testing of Participants’ Samples

The presented method has been applied on 28 autoptic samples from different organs obtained from 4 deceased PLWH who used opioids for palliative care during terminal disease.

All samples were successfully quantified for each drug. Concentrations are reported in Table 5 and expressed as ng/g. In order to evaluate incurred sample reanalysis, these samples have been re-analyzed, showing acceptable bias in compliance to EMA and FDA guidelines: 8.6% for MOR, 5.7% for O-MOR, 7.9% for H-MOR, 7.01% for O-COD, 5.9% for FENT and 7.7% for MTD. No sample containing H-COD was tested.

## 3. Discussion

The evaluation of opioid concentrations in tissues can give important insights about their distribution, tissue redistribution, possible tissue-specific effects and toxicity.

In this work, we described a multi-matrix, multiplexed method for the simultaneous quantification of the commonly used opioids in palliative care, which will be useful for studying drug distribution in autoptic specimens. The choice of the drugs to be included in this method was based on the most common opioid prescriptions [5,6] or pain management in terminal disease and on the drugs which were included in the treatment protocol from the “Last Gift” study. To assess the capability of this method to work in widely different matrices, we performed the validation considering several different tissues with widely different chemical compositions: heart, as a model for muscle tissue, subcutaneous fat, for fatty tissues, lung (alveolar tissue), liver (parenchymatous organ), intestine (one of main targets of opioid toxicity), kidney and plasma.

The results in terms of IS-nREC and IS-nME, both by post-extraction addition and standard curve slopes methods, confirmed the good robustness of this method. Nevertheless, it is important to note that the absolute REC and ME data, without the corrective effect of SIL-ISs, were widely variable: this highlights the importance of a rigorous internal standardization for a multi-matrix method as the one presented in this work. This aspect was particularly important considering the extremely variable chemical–physical composition of some of these tissues (e.g., fat, liver, and heart).

The developed method provided fast (7 min) and reliable results, confirming the eligibility for wider studies and, possibly, for medico-legal purposes. This multi-matrix validation, involving matrices with extremely different and variable composition, suggests the applicability of this method to other tissues with intermediate characteristics (e.g., brain tissue, smooth or striated muscles, etc.).

This method has been applied on seven sets of autoptic samples from four participants who lived with HIV. These samples confirmed the method’s capability of quantifying opioid concentrations in human samples on a wide range of tissue amounts (from 10 to 100 mg).

Participant 1 presented a pancreatic tumor and had taken O-COD 10 mg/day with a history of FENT use.

Participant 2 took 15 g of MOR as a part of his legal right-to-die option, as extremely high concentrations were found in all the compartments, with the higher ones in the left colon, heart, kidney and lung.

Participant 3 had a squamous cell carcinoma of tongue and had taken MTD and FENT (previously took MOR): opioid concentrations in this participant were more homogeneous among tissues and plasma.

Participant 4 presented with rectal cancer and was treated with H-MOR, showing higher concentrations in heart and lung as compared with other tissues.

Concerning the analyzed samples, higher opioids concentrations were observed in tissues than in plasma. Particularly, O-MOR and O-COD showed higher concentrations in kidney and liver than other tissues (>15–20 times greater) and blood plasma (>100 times greater).

The possible limits of this method consist in the difficulty of estimating the real recovery in tissue samples, where the drugs could be less available for extraction. Nevertheless, the double replicate testing of samples from participants showed satisfactory reproducibility (CV < 15%), even with tissue sections of variable weight, suggesting that this simple extraction technique could be still considered reliable.

To our knowledge, this is the first method able to quantify a wide panel of opioids in different tissue matrices; in addition, this study grants deeper insights on opioid distribution in tissues, paving the way for a better understanding of concentration-related organ-specific toxicity.

## 4. Materials and Methods

### 4.1. Chemicals and Reagents

The reference standard of FENT (purity 99.9%), MOR (purity 99.7%), MTD (purity 99.9%), O-MOR (purity 99.8%), O-COD (purity 99.8%), H-MOR (purity 99.6%), and H-COD (purity 99.8%) solutions in methanol (MeOH) were purchased from Sigma-Aldrich (Milan, Italy).

The compounds which were chosen as internal standards (IS) included (±)-FENT-D5 (purity 99.5%, isotopic purity 97.05%), MOR-D3 (purity 99.2%, isotopic purity 89.00%), (±)-MTD-D3 (purity 99.7%, isotopic purity 98.7%), O-MOR-D3 (purity 99.8%, isotopic purity 99.55%), O-COD-D3 (purity 99.9%, isotopic purity 93.30%), H-MTD-D3 (purity 99.7%, isotopic purity 89.70%), and H-COD-D3 (purity 99.8%, isotopic purity 86.49%) in MeOH were purchased from Sigma-Aldrich (Milan, Italy). HPLC-grade acetonitrile (ACN) and MeOH were purchased from VWR International (Radnor, PA, USA). HPLC grade water was produced with a Milli-DI system coupled with a Synergy 185 system by Millipore (Milan, Italy). Formic acid was purchased from Sigma-Aldrich (Milan, Italy). Blank plasma from healthy donors was kindly supplied by the Blood Bank of the “Città della Salute e della Scienza” of Turin, while blank tissue for method validation was obtained from meat for commercial use.

### 4.2. Preparation of Calibrators and Quality Control Samples

Standard solutions at the concentration of 1 mg/mL in MeOH were used to independently spike MeOH:H_2_O (70:30 *v*/*v*) to obtain the highest calibrating solution, which were used, in turn, for the preparation of the highest standard point of the calibration curve (STD 7). The same procedure was performed for the preparation of the quality control (QCs) solutions at 3 different concentrations: high, medium and low (QC H, M and L, respectively).

At each analytical session, other calibration standards (STD 6 to STD 1) were obtained by 1:3 (*v*/*v*) serial dilution of the STD 7 with MeOH:H_2_O (70:30 *v*/*v*). Then, these calibration standards were used to independently spike blank tissue aliquots to obtain calibration curves in different matrices: 100 µL of calibrating solutions were added to each aliquot of tissue (weight range 10–100 mg). Exact concentrations for each standard (STD), calibration ranges and QC values are reported in Table 2 and Table 3, respectively.

The type of matrices tested were as follows: heart, lung, kidney, liver, abdominal adipose tissue, intestine and plasma (post-mortem and pre-mortem, where available).

### 4.3. Sample Preparation

Before each analytical session, an internal standard (IS) working solution was prepared in MeOH:H_2_O (70:30 *v*/*v*) at the concentration of 10 ng/mL for MTD, 20 ng/mL for MOR, 5 ng/mL for FENT, O-COD, O-MOR, H-COD and H-MOR.

After thawing at room temperature, each sample was treated as follows: 40 µL of IS working solution and 100 µL of calibration standard were added to an amount of pestled weighed tissue.

Then, samples were vortex-mixed for at least 10 s and sonicated for 10 min.

Subsequently, in the extraction process, 360 µL of a precipitant solution (ACN:MeOH 50:50 *v*/*v*) was added, and then, samples were vortex-mixed for at least 10 s.

All samples were subsequently centrifuged at 10,000× *g* for 5 min, without brake, at 10 °C, and the obtained supernatants (400 µL) were transferred in glass shots and dried in a vacuum centrifuge at 50 °C for about 1.5 h. Finally, the dry extracts were dissolved first with 50 µL of H_2_O:MeOH 50:50 *v*/*v* and vortex-mixed; then, they were dissolved with 150 µL of H_2_O and vortex-mixed again; lastly, they were transferred in total recovery vials: 5 µL were injected in the chromatographic system.

### 4.4. LC-MS Analysis

The chromatographic separation was carried out with a LX50 UHPLC (Perkin Elmer, Milan, Italy) composed of an Integrity^®^ autosampler, a SPH1299^®^ Dual UHPLC Pump and a Mistral^®^ column oven. The chromatographic column was a Kinetex^®^ Biphenyl LC column, 2.1 × 100 mm, 2.6 μm (Phenomenex, Torrance, CA, USA) at 40 °C. The autosampler temperature was set at 15 °C.

The flow rate was settled at 0.4 mL/min with a gradient of two mobile phases: A (0.1% *v*/*v* formic acid in HPLC grade H_2_O) and B (0.1% formic acid *v*/*v* in MeOH and ACN 60:40 *v*/*v*). Briefly, the chromatographic gradient started with 10% Mobile Phase B up to 0.5 min. Then, it was increased linearly to 95% at 4.7 min and held at the same percentage up to 5.60 min. After, a decrease to 10% Mobile Phase B was applied from 5.65 min and held to the end of analysis.

The total runtime was 7 min. H_2_O:MeOH 95:5 vol:vol was used as weak washing solution, while H_2_O:ACN 30:70 vol:vol was adopted as strong washing solution. Two strong washing and two weak washing cycles (250 µL each) were applied, sequentially, after each injection.

Tandem mass spectrometry detection was carried out with a QSight 220^®^ (Perkin Elmer, Milan, Italy) tandem mass spectrometer with an electrospray ionization (ESI) interface.

The ESI source was set in positive ionization mode (ESI+) for all drugs.

“Zero-Air” (Dry air) was used as nebulizing and heating gas, while nitrogen was used as Drying and Collision gas: both these gasses were produced at high purity (>99.9%) with a Cinel Zefiro QS^®^ (Cinel, Vigonza, Italy).

The general mass parameters for positive ionization were as follows: electrospray voltage 5.0 kV; source temperature 350 °C; nebulizing gas flow 350 L/h; drying gas flow 130 L/h; Heated Surface-Induced Desolvation (HSID) temperature, 300 °C.

Two mass transitions yielding the highest sensitivity were selected for all drugs: the first was quantification trace and the second was ion trace. All masses are reported in Table 2.

### 4.5. Method Validation

Once we obtained enough separation between the different analytes and enough selectivity/specificity, the method underwent a full validation in compliance with EMA and FDA guidelines for bioanalytical methods validation [33,34]. The validation covered the following: specificity and selectivity, accuracy and precision, linearity and sensitivity, carry-over, recovery and matrix effect. Since the stability data were already available for these drugs [38], analyte stability was tested only in particular conditions associated with the method.

#### 4.5.1. Results of Specificity and Selectivity Test

The method’s specificity and selectivity were tested on 6–10 “blank” (without any trace of the analytes of interest) aliquots of each tissue after undergoing sample preparation. Good specificity and selectivity were interpreted as the absence of significant interfering peaks at the analytes retention times (with mean signal <20% of the LLOQ for the target analytes or 5% of the IS).

#### 4.5.2. Accuracy, Precision, Calibration, Limits of Quantification and Detection, Dilution Integrity

The accuracy and intra-day precision were evaluated in 5 intra-day replicates of each QC sample at 3 different concentrations. Inter-day precision was evaluated as the coefficient of variation (CV%) of the QC among 6 different validation sessions. The linearity of the calibration curve was evaluated in all the 6 sessions, following a linear fitting with 1/x weighing. The lower limit of quantification (LLOQ) and of detection (LOD) were considered as the lowest concentrations yielding signal-to-noise ratios of 5 and 3, respectively. Moreover, the bias% from the nominal value and CV% at the level of LLOQ had to be both <20%.

Finally, the dilution integrity was evaluated by quantifying in double replicate samples with a concentration twice higher than the highest point of the standard curve (STD 6) after a 3-fold dilution.

#### 4.5.3. Recovery

Recovery (REC) was estimated by comparing the signals of target analytes and their IS in 6 “blank” samples (without analytes) from different tissues spiked, after the extraction process, at the same concentration in vials of the QC samples (post-extraction addition) with the ones from the injection of QC samples (spiked before extraction). This was evaluated both as absolute REC and as IS-normalized-REC in order to assess the capability of the IS to mitigate the variability in REC data.

#### 4.5.4. Shelf-Life Results

The long- and short-term stability data at −20 and −80 °C were already extensively described in the literature [39,40,41,42]. However, a long-term stability study was performed up to 4 months at −80 °C on standards and QC samples (H and L) spiked in liver samples (from pig), since this tissue is expected to provide the highest potential for drug degradation, and in human plasma, in order to evaluate the feasibility of the samples’ shipment and medium-term storage. Moreover, the short-term stability bench-top (at 2, 4 and 24 h) and in the autosampler (at 24 h) was investigated, again from pig liver samples and human plasma.

In addition, 2 freeze and thaw cycles were performed.

#### 4.5.5. Matrix Effect

The percentage of matrix effect (ME) was calculated by comparing the signals of the target analytes and QC of “post-extraction” spiked QCs (see Recovery) with the ones from the direct injection of pure solvents with the same analytes concentrations [37]. Moreover, ME and REC were also evaluated by comparing the standard curve slopes prepared in all the different tissues tested (heart, lung, kidney, liver, abdominal adipose tissue, intestine and plasma) with the ones obtained from the same curves prepared in pure solvent, as suggested by Matuszewsky et al. [35,36].

#### 4.5.6. Carry-Over results

Carry-over was investigated by injecting blank plasma extracts after the injection of a sample extract prepared at a twice higher concentration than the highest standard sample (STD 6).

The absence of significant carry-over was defined as a signal in these samples < 20% of the LLOQ and 5% of the IS signal.

#### 4.5.7. Application and Statistical Analysis

This method was applied on autoptic specimens obtained in the context of the observational clinical study “Last Gift” (this study was approved by the UCSD Office of Human Research Protections Program, protocol 160563), which aims to study drug concentrations in tissues and plasma in people who lived with HIV (PLWH) and used opioids for palliative care during terminal disease. Chromatographic and mass spectrometry data were processed through Simplicity^®^ 3Q (Perkin Elmer, Milan, Italy) software. Drug concentrations were normalized by weight for each sample.

The Last Gift is a unique cohort that enrolls altruistic PLWH with a life-shortening illness who wish to participate in HIV cure research at the end-of-life (EOL), including tissue donation for a rapid research autopsy (completed within six hours of death), which greatly increases viable cell count, tissue and nucleic acid integrity, as well as maintaining the tissue distribution of drugs. The primary goal of the Last Gift study is to characterize the HIV reservoirs across various tissues and anatomic compartments. Opioid medications are used to relieve pain and suffering associated with terminal illness and might interact with HIV persistence. Thus, measuring levels of opioids in various tissues is a priority for the Last Gift study team.

#### 4.5.8. Samples Retesting

All the analyzed samples have been re-tested in the following analytical session. The CV% of these comparisons was calculated as a marker of “real-samples” precision.

## 5. Conclusions

The fast and simple method was fully validated in compliance with EMA and FDA guidelines for bioanalytical methods validation.

The obtained results gave evidence that the method could be useful for research purposes: it could be used for post-mortem pharmacological/toxicological studies, allowing to complement the lack of information in this field.

## Figures and Tables

**Table 1:** Overall evaluation of IS-nME and IS-nREC by comparison of the slopes as suggested by Matuszewsky et al. [35,36]. All deviation data are related to the curve prepared in pure solvent (reference calibration curve); the CV% is referred to the comparison between different tissues. M = calibration slope; k = calibration intercept.

	Morphine			Oxymorfone			Hydromorfone			Oxycodone			Hydrocodone			Fentanyl			Methadone		
	m	k	Dev. from Ref.	m	k	Dev. from Ref.	m	k	Dev. from Ref.	m	k	Dev. from Ref.	m	k	Dev. from Ref.	m	k	Dev. from Ref.	m	k	Dev. from Ref.
**Pure solvent** **(reference)**	1.030	0.007	**n.a.**	5.437	0.022	**n.a.**	3.752	0.027	**n.a.**	2.544	0.026	**n.a.**	3.980	−0.012	**n.a.**	4.451	−0.001	**n.a.**	2.236	0.002	**n.a.**
**Heart**	1.062	0.006	**3.1%**	4.994	0.046	**−8.1%**	3.730	0.007	**−0.6%**	2.510	0.006	**−1.3%**	4.042	−0.014	**1.6%**	4.422	0.005	**−0.7%**	2.442	0.024	**9.2%**
**Lung**	1.015	0.006	**−1.5%**	4.808	0.038	**−11.6%**	3.716	−0.004	**−1.0%**	2.488	0.015	**−2.2%**	4.050	−0.013	**1.8%**	4.422	0.002	**−0.7%**	2.406	0.014	**7.6%**
**Kidney**	1.011	0.007	**−1.8%**	5.26	−0.011	**−3.3%**	3.712	0.003	**−1.1%**	2.525	0.013	**−0.7%**	4.006	0.007	**0.7%**	4.420	0.003	**−0.7%**	2.35	0.017	**5.1%**
**Liver**	1.022	−0.001	**−0.8%**	5.62	0.042	**3.4%**	3.820	0.001	**1.8%**	2.678	−0.009	**5.3%**	3.982	0.016	**0.1%**	4.453	−0.006	**0.0%**	2.449	0.017	**9.5%**
**Intestine**	1.032	0.001	**0.2%**	5.476	0.046	**0.7%**	3.678	0.012	**−2.0%**	2.678	0.061	**5.3%**	4.053	−0.005	**1.8%**	4.416	0.001	**−0.8%**	2.36	0.009	**5.5%**
**Subcut. fat**	1.044	−0.012	**1.4%**	5.408	−0.053	**−0.5%**	3.598	0.003	**−4.1%**	2.385	0.024	**−6.3%**	3.932	−0.003	**−1.2%**	4.344	−0.005	**−2.4%**	2.203	0.015	**−1.5%**
**Plasma**	0.961	0.012	**−6.7%**	4.851	0.021	**−10.8%**	3.891	−0.031	**3.7%**	2.451	0.001	**−3.7%**	4.038	−0.022	**1.5%**	4.32	0.005	**−2.9%**	2.361	0.016	**5.6%**
	**Inter-tissue** **CV**		**Mean** **Dev.**	**Inter-tissue** **CV**		**Mean** **Dev.**	**Inter-tissue CV**		**Mean** **Dev.**	**Inter-tissue** **CV**		**Inter-tissue** **CV**	**Inter-tissue CV**		**Mean** **Dev.**	**Inter-tissue** **CV**		**Mean** **Dev.**	**Inter-tissue CV**		**Mean** **Dev.**
	**3.11%**		**−0.9%**	**6.17%**		**−4.3%**	**2.55%**		**−0.5%**	**4.37%**		**−0.5%**	**1.11%**		**0.9%**	**1.10%**		**−1.2%**	**3.50%**		**5.9%**

**Table 2:** For each drug are reported, in order: retention time (RT), the concentration at the highest standard point of the calibration curve (STD 7 to STD1/LLOQ), dwell times and mass transitions (precursor and product ions), with the corresponding entrance voltages and collision energies. All concentration data are referred to the initial plasma sample.

DRUGs	RT (min)	Calibration Range (ng)	[M+H]^+^ (*m*/*z*)	Dwell Time (ms)	Quantification Trace (*m*/*z*)	Entrance Voltage (V)	Collision Energy Second Product Ion Trace (eV)	Qualifier Trace (*m*/*z*)	Entrance Voltage (V)	Collision Energy First Ion Product Trace (eV)
**MRPH**	1.32	0.027–20	286.10	25	201.10	40	−32	165.10	40	−50
**MRPH-D_3_**	1.30	-	289.10	25	201.10	40	−33	165.10	40	−50
**O-MRPH**	1.47	0.020–15	302.10	25	227.10	30	−35	198.10	28	−60
**O-MRPH -D_3_**	1.45	-	305.10	25	230.10	30	−35	201.10	28	−60
**H-MRPH**	1.64	0.014–10	286.10	25	185.10	46	−40	128.10	47	−79
**H-MRPH -D_3_**	1.63	-	289.10	25	185.10	46	−40	128.10	47	−79
**O-COD**	2.15	0.020–15	316.20	25	212.10	30	−55	241.10	30	−55
**O-COD -D_3_**	2.14	-	319.20	25	215.10	30	−35	244.10	30	−55
**H-COD**	2.22	0.014–10	300.10	15	241.10	40	−34	199.10	40	−39
**H-COD -D_3_**	2.22	-	303.10	25	241.10	40	−34	199.10	40	−39
**FEN**	3.24	0.007–5	337.30	25	188.20	33	−31	105.10	34	−57
**FEN -D_5_**	3.23	-	342.30	25	188.20	33	−31	105.10	34	−57
**MET**	3.76	0.027–20	310.30	25	105.10	20	−40	91.00	20	−64
**MET -D_3_**	3.75	-	313.30	25	105.10	20	−40	92.10	20	−64

**Table 3:** Summary of the overall trueness, intra-day and inter-day precision for each drug in all the tested tissues. Lower limits of quantification (LOQ) and of detection (LOD).

Analyte	QC Level	Trueness (%)	Precision	
			Intra-Day (CV%)	Inter-Day (CV%)
**Morphine**	H (10 ng)	101.5	6.7	5.5
	M (1 ng)	98.4	4.4	7.5
	L (0.1 ng)	97.7	12.1	3.0
	LLOQ (0.027 ng)	111.2	11.3	8.2
	LOD (0.009 ng)	-	-	-
**Oxymorphone**	H (7.5 ng)	103.3	4.3	2.2
	M (0.75 ng)	104.6	6.5	3.0
	L (0.075 ng)	101.5	11.8	6.2
	LLOQ (0.020 ng)	107.8	12.1	10.2
	LOD (0.007 ng)	-	-	-
**Hydromorphone**	H (5 ng)	104.2	4.8	5.5
	M (0.5 ng)	99.8	7.9	7.1
	L (0.05 ng)	96.9	8.3	3.2
	LLOQ (0.014 ng)	108.2	10.8	12.1
	LOD (0.005 ng)	-	-	-
**Oxycodone**	H (7.5 ng)	101.1	7.8	1.5
	M (0.75 ng)	102.3	7.2	0.5
	L (0.075 ng)	96.3	10.9	8.7
	LLOQ (0.020 ng)	106.2	11.6	10.1
	LOD (0.007 ng)	-	-	-
**Hydrocodone**	H (5 ng)	102.2	5.6	2.7
	M (0.5 ng)	101.5	8.6	8.6
	L (0.05 ng)	97.6	12.5	6.8
	LLOQ (0.014 ng)	110.5	13.1	14.0
	LOD (0.005 ng)	-	-	-
**Fentanyl**	H (2.5 ng)	98.8	1.8	1.9
	M (0.25 ng)	96.8	2.2	2.2
	L (0.025 ng)	95.9	5.2	2.8
	LLOQ (0.007 ng)	98.2	9.3	9.1
	LOD (0.002 ng)	-	-	-
**Methadone**	H (10 ng)	97.5	5.2	0.7
	M (1 ng)	97.0	3.7	0.9
	L (0.1 ng)	93.6	7.0	3.6
	LLOQ (0.027 ng)	91.8	8.9	9.6
	LOD (0.009 ng)	-	-	-

**Table 4:** Summary of the evaluation of raw and IS-normalized REC and ME by post-extraction addition technique at 3 different concentrations in all the tested tissues.

Recovery and Matrix Effect by Post-Extraction Addition					
Analyte	QC Level	REC (%)	IS-nREC (%)	EM (%)	IS-nEM (%)
**Morphine**	H	88.8 (18.5)	91.8 (5.6)	−1.0 (21.9)	9.9 (5.3)
	M	92.9 (21.9)	99.0 (4.6)	−13.9 (22.6)	−5.0 (5.9)
	L	91.6 (26.5)	95.3 (10.0)	−19.7 (23.9)	4.8 (9.5)
**Oxymorphone**	H	90.2 (11.1)	101.2 (5.0)	0.1 (14.9)	0.3 (5.5)
	M	91.9 (14.9)	101.4 (3.7)	−12.5 (18.0)	1.2 (5.9)
	L	93.1 (17.7)	105.8 (10.3)	−15.5 (16.8)	−0.4 (5.1)
**Hydromorphone**	H	89.9 (13.0)	101.5 (4.2)	−1.5 (4.1)	−3.1 (2.4)
	M	94.3 (14.2)	100.4 (3.9)	−16.1 (10.6)	−3.0 (3.6)
	L	88.2 (20.2)	100.2 (8.2)	−2.9 (14.9)	8.8 (9.6)
**Oxycodone**	H	102.1 (24.9)	97.6 (8.4)	−22.5 (24.8)	−0.8 (9.9)
	M	97.7 (25.7)	100.1 (7.4)	−23.2 (17.4)	1.5 (5.8)
	L	95.5 (19.9)	97.9 (10.2)	−23.1 (19.2)	−6.9 (7.7)
**Hydrocodone**	H	108.8 (22.5)	99.7 (4.6)	−12.1 (25.3)	1.6 (5.7)
	M	99.6 (17.9)	101.5 (7.1)	−7.5(15.5)	2.5 (5.9)
	L	101.5 (19.7)	103.7 (6.6)	−2.3 (19.5)	1.0 (5.2)
**Fentanyl**	H	106.3 (32.5)	99.8 (1.7)	−33.2 (35.0)	−1.1 (1.1)
	M	102.2 (30.9)	99.3 (2.1)	−33.2 (19.9)	−1.3 (2.2)
	L	95.4 (29.1)	99.3 (4.8)	−23.8 (28.5)	0.3 (5.1)
**Methadone**	H	100.8 (30.3)	100.7 (5.1)	−28.8 (28.2)	−0.8 (4.2)
	M	104.9 (29.7)	101.8 (3.8)	−36.6 (19.4)	−2.6 (2.9)
	L	94.7 (26.3)	98.6 (7.2)	−30.3 (27.7)	6.1 (5.5)

**Table 5:** Summary of opioid concentrations in human autoptic samples.

	Participant 1						
	Morphine (ng/g) Mean (CV%)	Oxymorphone (ng/g) Mean (CV%)	Hydromorphone (ng/g) Mean (CV%)	Oxycodone (ng/g) Mean (CV%)	Hydrocodone (ng/g) Mean (CV%)	Fentanyl (ng/g) Mean (CV%)	Methadone (ng/g) Mean (CV%)
Heart	n.d.	33.4 (14.0)	n.d.	703.4 (18.2)	n.d.	n.d.	n.d.
Lung	n.d.	24.9 (10.7)	n.d.	1046.4 (6.9)	n.d.	n.d.	n.d.
Kidney	n.d.	380.7 (2.8)	n.d.	2580.6 (1.2)	n.d.	n.d.	n.d.
Liver	n.d.	317.5 (4.5)	n.d.	4421.7 (5.3)	n.d.	n.d.	n.d.
Left colon	n.d.	18.9 (0.8)	n.d.	455.7 (7.4)	n.d.	n.d.	n.d.
Abdominal adipose tissue	n.d.	3.9 (2.6)	n.d.	108.8 (3.4)	n.d.	n.d.	n.d.
Plasma (pre-mortem)	n.d.	2.0 (4.3)	n.d.	38.0 (6.7)	n.d.	n.d.	n.d.
	**Participant 2**						
	**Morphine (ng/g)** **Mean (CV%)**	**Oxymorphone (ng/g)** **Mean (CV%)**	**Hydromorphone (ng/g)** **Mean (CV%)**	**Oxycodone (ng/g)** **Mean (CV%)**	**Hydrocodone (ng/g)** **Mean (CV%)**	**Fentanyl (ng/g)** **Mean (CV%)**	**Methadone (ng/g)** **Mean (CV%)**
Heart	75,753 (5.7)	n.d.	n.d.	n.d.	n.d.	n.d.	n.d.
Lung	13,119 (13.6)	n.d.	n.d.	n.d.	n.d.	n.d.	n.d.
Kidney	11,739 (15.1)	n.d.	n.d.	n.d.	n.d.	n.d.	n.d.
Liver	10,166 (2.8)	n.d.	n.d.	n.d.	n.d.	n.d.	n.d.
Left colon	413,709 (3.5)	n.d.	n.d.	n.d.	n.d.	n.d.	n.d.
Abdominal adipose tissue	510 (4.2)	n.d.	n.d.	n.d.	n.d.	n.d.	n.d.
Plasma (post-mortem)	4013 (7.8)	n.d.	n.d.	n.d.	n.d.	n.d.	n.d.
	**Participant 3**						
	**Morphine (ng/g)** **Mean (CV%)**	**Oxymorphone (ng/g)** **Mean (CV%)**	**Hydromorphone (ng/g)** **Mean (CV%)**	**Oxycodone (ng/g)** **Mean (CV%)**	**Hydrocodone (ng/g)** **Mean (CV%)**	**Fentanyl (ng/g)** **Mean (CV%)**	**Methadone (ng/g)** **Mean (CV%)**
Heart	n.d.	n.d.	n.d.	n.d.	n.d.	60.5 (2.6)	569 (1.1)
Lung	n.d.	n.d.	n.d.	n.d.	n.d.	80.7 (0.5)	1699 (15.9)
Kidney	323.2 (16.3)	n.d.	n.d.	n.d.	n.d.	57.1 (19.5)	710 (18.2)
Liver	n.d.	n.d.	n.d.	n.d.	n.d.	96 (3.3)	853 (1.3)
Left colon	n.d.	n.d.	n.d.	n.d.	n.d.	14.1 (13.4)	171 (8.0)
Abdominal adipose tissue	n.d.	n.d.	n.d.	n.d.	n.d.	45.3 (1.2)	239 (4.4)
Plasma (post-mortem)	n.d.	n.d.	n.d.	n.d.	n.d.	10.6 (1.3)	181 (4.9)
	**Participant 4**						
	**Morphine (ng/g)** **Mean (CV%)**	**Oxymorphone (ng/g)** **Mean (CV%)**	**Hydromorphone (ng/g)** **Mean (CV%)**	**Oxycodone (ng/g)** **Mean (CV%)**	**Hydrocodone (ng/g)** **Mean (CV%)**	**Fentanyl (ng/g)** **Mean (CV%)**	**Methadone (ng/g)** **Mean (CV%)**
Heart	n.d.	n.d.	221.6 (11.1)	n.d.	n.d.	n.d.	n.d.
Lung	n.d.	n.d.	221.6 (15.8)	n.d.	n.d.	n.d.	n.d.
Kidney	n.d.	n.d.	134.4 (1.4)	n.d.	n.d.	n.d.	n.d.
Liver	n.d.	n.d.	39.8 (9.6)	n.d.	n.d.	n.d.	n.d.
Left colon	n.d.	n.d.	37.7 (8.6)	n.d.	n.d.	n.d.	n.d.
Abdominal adipose tissue	n.d.	n.d.	54.6 (6.4)	n.d.	n.d.	n.d.	n.d.
Plasma (pre-mortem)	n.d.	n.d.	9.0 (2.3)	n.d.	n.d.	n.d.	n.d.

## Data Availability

Data will be provided on request.

## Supplementary Materials

The following supporting information can be downloaded at: https://www.mdpi.com/article/10.3390/ph16060903/s1, Supplementary Table S1: Short-term and long-term stability results.
