# Supplementary material for: A Novel UHPLC-MS/MS Method for the Quantification of Seven Opioids in Different Human Tissues
Source: Pharmaceuticals (Basel). 2023 Jun 19;16(6):903. doi: 10.3390/ph16060903 (PMC10300969; doi:10.3390/ph16060903)
Supplement: Supplementary file 1 [file pharmaceuticals-16-00903-s001.zip › pharmaceuticals-2445517-supplementary.pdf]

**Supplementary Table S1.** Short-term and long-term stability results.

| MRPH | | | | |
|---|---|---|---|---|
| **Condition** | **Tissue** | **Timing** | **QC H (% acc.)** | **QC L (% acc.)** |
| Long-term -80°C | Liver | 4 months | 96.3 | 95.4 |
| | Plasma | 4 months | 97.8 | 92.3 |
| Bench-top | Liver | 2 h | 104.4 | 113.6 |
| | | 4 h | 92.5 | 112.1 |
| | | 24 h | 110.9 | 89.7 |
| | Plasma | 2 h | 102.8 | 109.9 |
| | | 4 h | 107.4 | 108.5 |
| | | 24 h | 104.0 | 94.0 |
| Autosampler | Liver | 24 h | 102.0 | 97.9 |
| | Plasma | 24 h | 93.3 | 95.3 |
| Freeze-thaw | Liver | 2 cycles | 91.3 | 92.4 |
| | Plasma | 2 cycles | 94.2 | 95.3 |
| **O-MRPH** | | | | |
| **Condition** | **Tissue** | **Timing** | **QC H (% acc.)** | **QC L (% acc.)** |
| Long-term -80°C | Liver | 4 months | 95.1 | 91.7 |
| | Plasma | 4 months | 96.3 | 93.6 |
| Bench-top | Liver | 2 h | 99.0 | 105.0 |
| | | 4 h | 92.9 | 97.1 |
| | | 24 h | 88.9 | 102.6 |
| | Plasma | 2 h | 98.6 | 97.1 |
| | | 4 h | 103.1 | 100.8 |
| | | 24 h | 110.8 | 99.4 |
| Autosampler | Liver | 24 h | 93.3 | 86.6 |
| | Plasma | 24 h | 108.4 | 88.6 |
| Freeze-thaw | Liver | 2 cycles | 92.6 | 95.2 |
| | Plasma | 2 cycles | 93.7 | 89.9 |
| **H-MRPH** | | | | |
| **Condition** | **Tissue** | **Timing** | **QC H (% acc.)** | **QC L (% acc.)** |
| Long-term -80°C | Liver | 4 months | 98.2 | 96.4 |
| | Plasma | 4 months | 97.5 | 98.6 |
| Bench-top | Liver | 2 h | 104.7 | 101.8 |
| | | 4 h | 107.2 | 108.4 |
| | | 24 h | 90.3 | 100.0 |
| | Plasma | 2 h | 98.2 | 101.8 |
| | | 4 h | 98.3 | 108.4 |

| | | | | |
|---|---|---|---|---|
| | | 24 h | 108.5 | 99.4 |
| Autosampler | Liver | 24 h | 94.5 | 97.4 |
| | Plasma | 24 h | 108.4 | 94.9 |
| Freeze-thaw | Liver | 2 cycles | 90.1 | 89.8 |
| | Plasma | 2 cycles | 94.2 | 92.0 |
| | | **O-COD** | | |
| **Condition** | **Tissue** | **Timing** | **QC H (% acc.)** | **QC L (% acc.)** |
| Long-term -80°C | Liver | 4 months | 101.2 | 96.3 |
| | Plasma | 4 months | 98.3 | 94.7 |
| Bench-top | Liver | 2 h | 103.3 | 106.7 |
| | | 4 h | 103.8 | 105.2 |
| | | 24 h | 95.7 | 111.8 |
| | Plasma | 2 h | 105.3 | 97.7 |
| | | 4 h | 103.6 | 98.2 |
| | | 24 h | 97.0 | 94.3 |
| Autosampler | Liver | 24 h | 95.9 | 100.0 |
| | Plasma | 24 h | 94.6 | 87.9 |
| Freeze-thaw | Liver | 2 cycles | 95.3 | 96.0 |
| | Plasma | 2 cycles | 97.2 | 98.1 |
| | | **H-COD** | | |
| **Condition** | **Tissue** | **Timing** | **QC H (% acc.)** | **QC L (% acc.)** |
| Long-term -80°C | Liver | 4 months | 99.8 | 94.1 |
| | Plasma | 4 months | 97.7 | 98.3 |
| Bench-top | Liver | 2 h | 108.0 | 96.7 |
| | | 4 h | 89.5 | 105.8 |
| | | 24 h | 95.0 | 109.2 |
| | Plasma | 2 h | 98.4 | 112.3 |
| | | 4 h | 96.8 | 106.4 |
| | | 24 h | 97.3 | 101.6 |
| Autosampler | Liver | 24 h | 101.7 | 112.2 |
| | Plasma | 24 h | 91.8 | 101.7 |
| Freeze-thaw | Liver | 2 cycles | 94.1 | 95.6 |
| | Plasma | 2 cycles | 92.3 | 93.1 |
| | | **FENTANYL** | | |
| **Condition** | **Tissue** | **Timing** | **QC H (% acc.)** | **QC L (% acc.)** |
| Long-term -80°C | Liver | 4 months | 93.0 | 99.1 |
| | Plasma | 4 months | 95.9 | 94.2 |
| Bench-top | Liver | 2 h | 98.6 | 91.9 |

| | | | | |
|---|---|---|---|---|
| | | 4 h | 101.7 | 92.3 |
| | | 24 h | 103.7 | 87.1 |
| | Plasma | 2 h | 99.4 | 103.5 |
| | | 4 h | 103.5 | 112.5 |
| | | 24 h | 97.3 | 100.0 |
| Autosampler | Liver | 24 h | 100.1 | 87.1 |
| | Plasma | 24 h | 95.3 | 94.5 |
| Freeze-thaw | Liver | 2 cycles | 93.0 | 91.5 |
| | Plasma | 2 cycles | 95.7 | 96.1 |
| | | **METHADONE** | | |
| **Condition** | **Tissue** | **Timing** | **QC H (% acc.)** | **QC L (% acc.)** |
| Long-term -80°C | Liver | 4 months | 100.3 | 96.0 |
| | Plasma | 4 months | 98.1 | 91.6 |
| Bench-top | Liver | 2 h | 99.8 | 88.4 |
| | | 4 h | 88.2 | 87.3 |
| | | 24 h | 97.3 | 103.2 |
| | Plasma | 2 h | 103.0 | 104.3 |
| | | 4 h | 112.6 | 105.1 |
| | | 24 h | 99.0 | 99.1 |
| Autosampler | Liver | 24 h | 101.4 | 107.5 |
| | Plasma | 24 h | 94.9 | 88.7 |
| Freeze-thaw | Liver | 2 cycles | 94.5 | 96.2 |
| | Plasma | 2 cycles | 98.3 | 95.1 |
